# Gastric Juice miR-106a-5p as a Non-Invasive Biomarker of Neuroinflammation and Neurodegeneration: A Prospective Observational Study

**DOI:** 10.3390/diseases14060187

**Published:** 2026-05-25

**Authors:** Sabrina Birsan, Iulian Roman-Filip, Mihai Rusu, Fratila Anca, Adrian Boicean, Pogony Sebastian, Grama Blanca, Corina Roman-Filip

**Affiliations:** 1 Faculty of Medicine, University Lucian Blaga, 550024 Sibiu, Romania; sabrinaandreea.marinca@ulbsibiu.ro (S.B.); dr.mihairusu@yahoo.com (M.R.); anca.fratila@ulbsibiu.ro (F.A.); adrian.boicean@ulbsibiu.ro (A.B.); pogonyi.sebastian@gmail.com (P.S.); corina.roman@ulbsibiu.ro (C.R.-F.); 2 Clinical Department, Faculty of Medicine, University of Medicine ‘George Emil Palade’, 540139 Targu Mure, Romania; 3 Faculty of Psychology; University Lucian Blaga of Sibiu, 550024 Sibiu, Romania; blanca.grama@ulbsibiu.ro

**Keywords:** neuroinflammation, gut microbiota, MIRNAs

## Abstract

Background: Neuroinflammation is a key contributor to the progression of several neurodegenerative disorders, including Alzheimer’s disease, stroke, and small vessel disease. Emerging evidence highlights the role of circulating microRNAs (miRNAs) as non-invasive biomarkers of neuroinflammation and neuronal injury. miR-106a-5p, a member of the miR-17~92 cluster, is known to regulate inflammation, apoptosis, and vascular function. While typically studied in plasma or cerebrospinal fluid, gastric juice miRNAs represent a novel and underexplored source for biomarker discovery within the gut–brain axis. This exploratory study aimed to investigate the association between gastric juice miR-106a-5p expression and markers of neuroinflammation, including C-reactive protein (CRP), lactate dehydrogenase (LDH), and imaging-based evidence of neurodegeneration. Methods: A prospective, observational study was conducted on 38 participants (22 with neurodegenerative pathology and 16 healthy controls). Gastric juice samples were analyzed for miR-106a-5p using RT-qPCR, normalized to U6 snRNA. ΔCt values were used to determine relative expression. Statistical analyses included *t*-tests/Wilcoxon tests, ROC curve analysis, and correlation testing, with significance set at *p* < 0.05. Results: Patients with neurodegenerative changes exhibited significantly lower gastric miR-106a-5p expression compared to controls (*p* = 0.044). Elevated CRP and LDH levels were associated with higher ΔCt values (indicating lower expression), with *p*-values of 0.019 and 0.023, respectively. ROC analysis showed moderate diagnostic accuracy (AUC = 0.701) for miR-106a in identifying neurodegenerative status. miR-106a levels also correlated inversely with carotid intima-media thickness and brain MRI abnormalities, also reduced gastric miR-106a-5p expression is associated with systemic inflammation and neuroimaging evidence of neurodegeneration. Conclusions: While causality cannot be inferred, these findings suggest that gastric miR-106a may serve as a promising non-invasive biomarker within the gut–brain axis framework. Further longitudinal and mechanistic studies are warranted to validate its clinical utility and explore its potential role in monitoring neuroinflammatory conditions.

## 1. Introduction

Neuroinflammation is increasingly recognized as a central mechanism in the pathogenesis of numerous neurological disorders, including Alzheimer’s disease, Parkinson’s disease, multiple sclerosis, and ischemic stroke. It involves activation of glial cells, particularly microglia and astrocytes, disruption of blood–brain barrier integrity, and release of pro-inflammatory mediators, ultimately contributing to neuronal dysfunction, synaptic damage, and cognitive decline [1,2]. Although neuroinflammatory responses may initially exert protective effects, chronic or dysregulated activation is considered a major driver of progressive neurodegeneration [1,2]. At present, the evaluation of neuroinflammatory and neurodegenerative disorders relies predominantly on clinical assessment and neuroimaging techniques, including magnetic resonance imaging (MRI) and computed tomography (CT). While these imaging modalities are essential for identifying structural brain abnormalities, they may have limitations in detecting early inflammatory changes and do not provide information regarding systemic inflammatory signaling or molecular alterations associated with disease progression [1,2,3]. Consequently, increasing attention has been directed toward the identification of novel non-invasive biomarkers capable of reflecting both peripheral and central nervous system pathology.

MicroRNAs (miRNAs) have emerged as promising biomarker candidates because of their remarkable stability in biological fluids and their involvement in multiple regulatory pathways associated with inflammation, endothelial dysfunction, apoptosis, oxidative stress, and neurodegeneration [3,4,5,6]. Among these, miR-106a-5p, a member of the miR-17~92 cluster, participates in several biological processes including angiogenesis, inflammatory regulation, neurovascular remodeling, and cellular survival pathways [3,4,5,6]. Previous studies have linked dysregulation of miR-106a-5p with endothelial injury, oxidative stress, and microglial activation relevant to central nervous system (CNS) pathology [3,4,5,6]. In particular, Du et al. demonstrated altered miR-106a-5p expression in both serum and cerebrospinal fluid in patients with acute cerebral infarction, with significant correlations between miR-106a-5p levels and inflammatory cytokines, supporting its potential role as a biomarker of neuroinflammatory injury [3].

Although most studies investigating neurological miRNAs have focused on plasma or cerebrospinal fluid, growing interest has emerged in alternative biofluids that may better reflect systemic and gut-related molecular alterations. Gastric juice represents an underexplored but potentially valuable source of miRNA biomarkers. Importantly, miRNAs in gastric juice are stabilized by extracellular vesicles and Argonaute protein complexes, allowing reliable detection even under highly acidic conditions [7,8,9,10]. Furthermore, the gut–brain axis is increasingly recognized as a bidirectional communication network involving immune, endocrine, neural, and microbial signaling pathways capable of modulating neuroinflammation and brain function [11]. Within this framework, gastric-derived miRNAs may provide unique insights into systemic inflammatory activity and gut-associated signaling relevant to neurodegenerative disease.

Previous studies have demonstrated that miR-106a-5p can be detected in gastric-related biological samples and may be influenced by gastrointestinal inflammation and systemic inflammatory conditions [7,12,13,14] Cui et al. reported dysregulated gastric juice miRNA expression profiles in gastric pathology, suggesting that gastric miRNAs may serve as clinically relevant non-invasive biomarkers [7]. In addition, miR-106a-5p dysregulation has been associated with inflammatory signaling, endothelial dysfunction, vascular remodeling, and neurovascular injury [15,16,17,18]. Preliminary evidence suggests that alterations in gastric miRNA profiles may indirectly reflect processes linked to neuroinflammation, vascular dysfunction, and cellular injury [11,12,13,14,15]. Based on these observations, we hypothesized that gastric juice miR-106a-5p expression is reduced in individuals presenting neurodegenerative changes visible on MRI compared with healthy controls. miR-106a-5p was selected because of its previously reported involvement in inflammatory regulation, endothelial dysfunction, apoptosis, oxidative stress responses, and neurovascular injury [15,16,17,18]. Altered miR-106a-5p expression has been described in ischemic stroke, vascular inflammation, and neurodegenerative conditions, suggesting that this miRNA may participate in biological pathways linking systemic inflammation and CNS injury [3,16,17,18].

We further hypothesized that reduced miR-106a-5p expression would be associated with elevated inflammatory markers, including C-reactive protein (CRP) and lactate dehydrogenase (LDH), as well as markers of vascular pathology such as increased carotid intima-media thickness (cIMT) [19,20,21,22]. Recent evidence suggests that miRNAs may function as intercellular signaling molecules within the gut–brain axis through extracellular vesicle-mediated communication [10,22]. Gastric epithelial and immune cells may release miRNAs in response to local inflammation, oxidative stress, or metabolic disturbances, thereby contributing to systemic inflammatory signaling [10,22]. These circulating inflammatory mediators and miRNA-containing vesicles may subsequently influence blood–brain barrier permeability and microglial activation, thereby participating in neuroinflammatory processes [1,2,15,22]. Within this context, gastric-derived miRNAs may represent accessible surrogate markers of systemic and gut-associated inflammatory activity relevant to CNS dysfunction. Therefore, the present exploratory observational study aimed to investigate the association between gastric juice miR-106a-5p expression and markers of neuroinflammation, vascular dysfunction, and neurodegenerative pathology. While the findings are intended to highlight biologically relevant associations, causality cannot be inferred from the present study design.

## 2. Materials and Methods

A prospective, observational study was conducted at the Gastroenterology Department of Sibiu Clinical Emergency Hospital in collaboration with the Molecular Biology Laboratory of Lucian Blaga University of Sibiu, Romania. The study received ethical approval, and all participants provided informed consent in accordance with the Declaration of Helsinki. A total of 38 participants were enrolled, including 22 patients with confirmed neurodegenerative pathology and 16 healthy controls. Inclusion criteria were adults aged 18 years or older with a diagnosis of neurodegenerative disease or no known neurological pathology. Given the exploratory nature of the study and the limited sample size, participants with different neurodegenerative conditions were analyzed as a single group. This approach was based on shared underlying pathophysiological mechanisms, including neuroinflammation, vascular dysfunction, and blood–brain barrier impairment, which represent common features across these disorders and MRI modifications. Participants were excluded if they had brain metastases, primary central nervous system tumors, or autoimmune CNS diseases. Clinical evaluation included neurological assessment using the Mini-Mental State Examination (MMSE) and neuroimaging (MRIscans) to detect signs of neurodegeneration, also the inflammatory status was assessed using C-reactive protein (CRP) and lactate dehydrogenase (LDH) levels. Additional variables collected included age, gender, and carotid intima-media thickness (cIMT) to evaluate vascular involvement.

Gastric juice samples (500 µL) were collected from each participant for miRNA analysis. Total RNA, including small RNAs, was extracted using TRIzol reagent (Invitrogen/Thermo Fisher Scientific, Carlsbad, CA, USA), and miRNA quantification was performed using the Qubit miRNA Assay Kit. Reverse transcription and quantitative real-time PCR (RT-qPCR) were carried out using TaqMan MicroRNA Reverse Transcription Kit Thermo Fisher Scientific (under the Applied Biosystems™ brand), Carlsbad, CA, USA, assays specific for miR-106a-5p, with U6 small nuclear RNA (snRNA) used as the endogenous control for normalization. U6 snRNA was used as the endogenous reference control for normalization. Although U6 is commonly used in cellular miRNA RT-qPCR studies, its stability in extracellular biofluids may be variable. Therefore, this normalization strategy should be interpreted cautiously, and future validation studies should include extracellular spike-in controls and/or validated stable endogenous miRNAs for gastric juice samples.

miR-106a expression levels were analyzed using the ΔCt method, where a higher ΔCt value indicates lower miRNA expression. Statistical analyses were conducted using IBM- SPSS version 22.00 (IBM Corp., Armonk, NY, USA) and R software, software, version 4.4.2 (R Foundation for Statistical Computing, Vienna, Austria). The Shapiro–Wilk test was used to assess normality, and depending on the data distribution, either *t*-tests or Wilcoxon rank-sum tests were applied for group comparisons. Receiver operating characteristic (ROC) curve analysis was used to evaluate diagnostic performance, and point-biserial correlation was calculated to assess associations. A *p*-value of less than 0.05 was considered statistically significant.

## 3. Results

The study group, composed of patients with MRI-confirmed neurological abnormalities, exhibited significantly altered clinical and biochemical profiles compared with the control group. Due to the limited sample size and the heterogeneity of neurological conditions included in the study, subgroup analyses according to specific neurological diagnoses were not performed. The study group was significantly older than the control group (median age 67.5 years, IQR 61–73 vs. 55 years, IQR 52–59; *p* = 0.001) and demonstrated lower cognitive performance, reflected by significantly reduced MMSE scores (median 19 vs. 24.5; *p* = 0.003). However, although age was significantly associated with disease status, no significant correlation was identified between age and miR-106a-5p ΔCt values (Spearman r = 0.013, *p* = 0.937), suggesting that the observed alterations in miR-106a-5p expression were not primarily age-dependent.

Gender distribution and area of residence did not differ significantly between groups. In contrast, vascular and neuroimaging markers demonstrated marked differences. Increased carotid intima-media thickness (cIMT > 1.3 mm) was observed in 77.3% of patients in the study group compared with 31.2% of controls (*p* = 0.005). Furthermore, all patients in the study group demonstrated pathological MRI findings, compared with 62.5% of controls (*p* = 0.003).

Metabolic and inflammatory biomarkers were also significantly elevated in the study group. Total lipid levels (median 900 mg/dL vs. 550 mg/dL; *p* < 0.001), triglycerides (350 mg/dL vs. 160 mg/dL; *p* < 0.001), LDL cholesterol (178.5 mg/dL vs. 149 mg/dL; *p* = 0.002), CRP (23.5 mg/L vs. 2.5 mg/L; *p* < 0.001), and LDH (270 U/L vs. 152.5 U/L; *p* < 0.001) were all significantly higher in patients with neurological abnormalities. In contrast, HDL cholesterol and ALP values did not differ significantly between groups.

Importantly, log_10_-transformed gastric juice miR-106a-5p expression levels were significantly lower in the study group compared with controls (median 3.15, IQR 2.15–4.18 vs. 4.94, IQR 3.12–5.35; *p* = 0.044), suggesting a potential association between miR-106a-5p downregulation and the presence of neurodegenerative and neuroinflammatory abnormalities (Table 1). Overall, patients with imaging-detected neurological abnormalities exhibited a distinct inflammatory, metabolic, and vascular profile accompanied by reduced gastric miR-106a-5p expression, supporting its potential role as a non-invasive biomarker associated with neuroinflammation and neurovascular injury.

**Figure 1:**
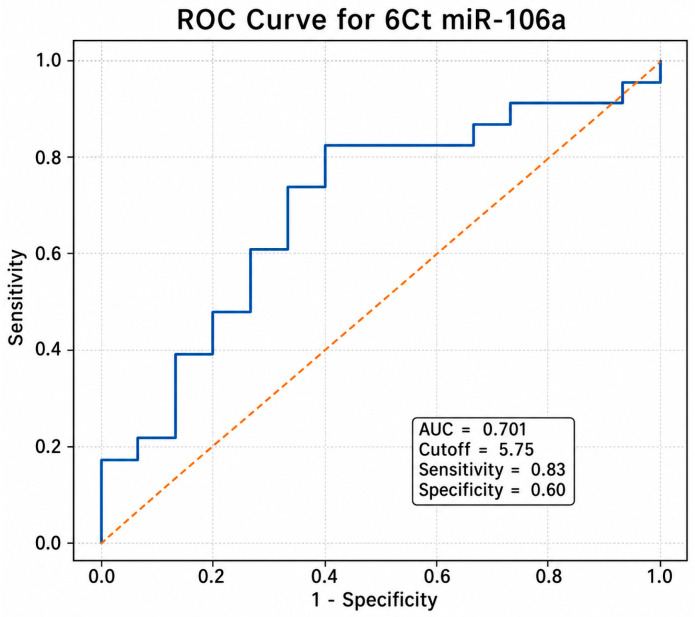
ROC curve analysis of ΔCt miR-106a expression for discrimination between study and control groups. Receiver operating characteristic (ROC) curve analysis demonstrating the diagnostic performance of ΔCt miR-106a expression for differentiating patients with neurological abnormalities from controls. The analysis yielded an area under the curve (AUC) of 0.701, indicating moderate discriminatory ability. The optimal cutoff value was 5.75, corresponding to a sensitivity of 83% and a specificity of 60%. Higher ΔCt values, reflecting lower miR-106a expression, were associated with the presence of neurodegenerative and neuroinflammatory abnormalities.

ROC curve analysis demonstrated that gastric juice-derived miR-106a-5p exhibits moderate discriminatory ability for differentiating patients with neurodegenerative abnormalities from healthy controls (AUC = 0.70). The optimal cutoff value was 5.75, corresponding to a sensitivity of 83% and a specificity of 60%. These findings suggest that gastric miR-106a-5p may represent a promising exploratory non-invasive biomarker within the gut–brain axis framework (Figure 1).

Among the evaluated biomarkers, CRP demonstrated the highest diagnostic accuracy (AUC = 0.95), followed by LDH (AUC = 0.82), both outperforming miR-106a-5p in distinguishing pathological cases from controls. However, unlike CRP and LDH, which primarily reflect systemic inflammation and tissue injury, miR-106a-5p may provide complementary biological information related to molecular regulatory pathways involved in neuroinflammation, endothelial dysfunction, apoptosis, neurovascular injury, and gut–brain axis signaling.

Therefore, gastric miR-106a-5p should not be interpreted as a replacement for conventional inflammatory biomarkers, but rather as a potential adjunctive biomarker reflecting broader neurovascular and inflammatory dysregulation. Although these findings support the potential relevance of miR-106a-5p as a minimally invasive biomarker, its diagnostic performance remains moderate and should be interpreted cautiously given the relatively small sample size and exploratory nature of the study. Further validation in larger, independent, and longitudinal cohorts is necessary before any clinical applicability or prognostic utility can be established.

**Figure 2:**
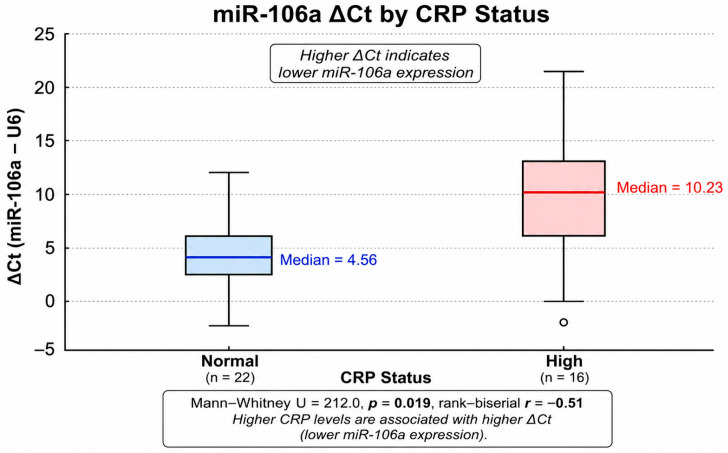
miR-106a Expression is significantly reduced in individuals with elevated CPR Levels. Boxplot representation of ΔCt values (miR-106a − U6) according to C-reactive protein (CRP) status. Higher ΔCt values indicate lower miR-106a expression. Patients with elevated CRP levels demonstrated significantly higher ΔCt values compared with individuals with normal CRP levels, indicating reduced miR-106a expression in the presence of systemic inflammation. Median ΔCt values were 10.23 in the high CRP group and 4.56 in the normal CRP group. Statistical analysis using the Mann–Whitney U test demonstrated a significant difference between groups (U = 212.0, *p* = 0.019). Rank-biserial correlation analysis revealed a moderate inverse association between elevated CRP levels and miR-106a expression (r = −0.51), suggesting that increased systemic inflammation is associated with reduced gastric miR-106a-5p expression.

We report that gastric juice miR-106a-5p expression, assessed using ΔCt values, differed significantly between individuals with normal and elevated CRP levels. Patients with increased CRP levels exhibited substantially higher ΔCt values (median = 10.23) compared with individuals presenting normal CRP values (median = 4.56), corresponding to reduced miR-106a-5p expression in the presence of systemic inflammation. This difference was statistically significant, as demonstrated by the Mann–Whitney U test (U = 212.0, *p* = 0.019). In addition, rank-biserial correlation analysis revealed a moderate-to-strong inverse association between CRP status and miR-106a-5p expression (r = −0.51), suggesting that increased systemic inflammatory activity is associated with reduced gastric miR-106a-5p expression (Figure 2). These findings support a potential relationship between miR-106a-5p downregulation and systemic inflammatory processes involved in neurovascular and neurodegenerative pathology.

**Figure 3:**
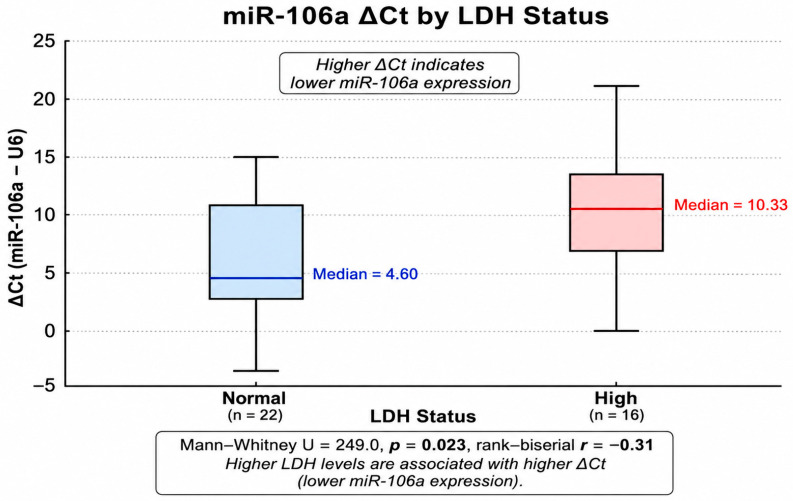
Association between miR-106a expression and LDH status. Boxplot representation of ΔCt values (miR-106a − U6) according to lactate dehydrogenase (LDH) status. Higher ΔCt values indicate lower miR-106a expression. Patients with elevated LDH levels demonstrated significantly higher ΔCt values compared with individuals with normal LDH levels, corresponding to reduced miR-106a expression. Median ΔCt values were 10.33 in the high LDH group and 4.60 in the normal LDH group. Statistical analysis was performed using the Mann–Whitney U test, revealing a significant difference between groups (U = 249.0, *p* = 0.023). Rank-biserial correlation analysis demonstrated a moderate inverse association between elevated LDH and miR-106a expression (r = −0.31).

The study results emphasized a significant association between reduced miR-106a-5p expression and elevated LDH levels. The expression of miR-106a-5p, assessed using ΔCt values, differed significantly between individuals with normal and elevated LDH levels, also patients with high LDH levels demonstrated substantially higher ΔCt values (median = 10.33) compared with those presenting normal LDH levels (median = 4.60), indicating reduced miR-106a expression in the presence of increased cellular injury and metabolic stress. This difference was statistically significant, as demonstrated by the Mann–Whitney U test (U = 249.0, *p* = 0.023). In addition, rank-biserial correlation analysis revealed a modest inverse association between LDH status and miR-106a-5p expression (r = −0.31), suggesting that reduced gastric miR-106a-5p expression is associated with elevated LDH levels. These findings may indicate a potential relationship between miR-106a downregulation, systemic cellular injury, and neurodegenerative pathological processes (Figure 3).

**Figure 4:**
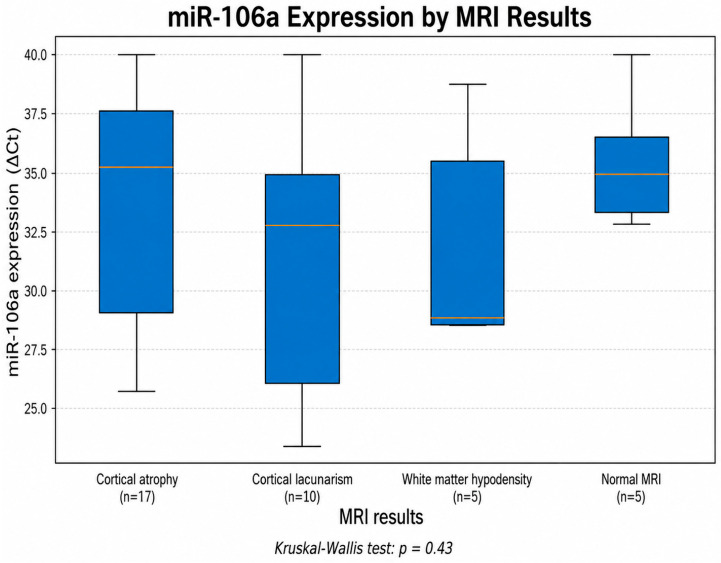
Gastric juice miR-106a-5p expression according to MRI-defined neurodegenerative abnormalities. Boxplot representation of gastric juice miR-106a ΔCt values across MRI-defined subgroups including cortical atrophy, cortical lacunarism, white matter hypodensity, and normal MRI findings. Central lines represent medians, boxes indicate interquartile ranges, and whiskers represent minimum and maximum values. Differences between groups were analyzed using the Kruskal–Wallis test because of non-normal distribution and unequal subgroup sizes (*p* = 0.43).

Patients with hypodensity in white matter show intermediate levels of miRNA, while those with lacunar infarctions have the lowest median miR-106a-5p levels. Furthermore, there is notable variability in the lacunar infarction group, as indicated by the large interquartile range, also, an outlier was noted in the hypodensity group. Although the overall comparison between MRI-defined subgroups did not reach statistical significance (Kruskal–Wallis test, *p* = 0.43), a visible trend toward altered miR-106a-5p expression was observed in patients with neurodegenerative and cerebrovascular abnormalities. These findings may suggest a potential relationship between reduced gastric miR-106a-5p expression and structural brain alterations associated with neuroinflammation and cerebral small vessel disease. However, the limited sample size and subgroup heterogeneity likely reduced the statistical power of the analysis (Figure 4).

We note that the consistent reduction in miR-106a-5p expression observed in individuals with elevated CRP and LDH levels, as well as those presenting with abnormal brain MRI findings—including cortical atrophy, lacunar infarctions, and white matter hypodensities—suggests a potential association between systemic inflammation, metabolic stress, and structural brain pathology. MiR-106a-5p is known to regulate key processes such as inflammation, apoptosis, and cell cycle progression, its downregulation in the context of elevated CRP and LDH may reflect a heightened inflammatory state and tissue damage, both peripherally and within the central nervous system. The overlap between these biochemical markers and imaging findings strengthens the hypothesis that reduced miR-106a -5p expression could serve as a surrogate marker for neuroinflammatory or neurodegenerative conditions. Also, from a clinical point of view, miR-106a-5p may have value as a non-invasive biomarker for identifying individuals at risk of cerebral small vessel disease or neurodegeneration, particularly when used alongside inflammatory markers and neuroimaging. The detection of miR-106a-5p in gastric juice adds a novel and accessible dimension to this biomarker’s potential, suggesting that alterations in gut-derived miRNA profiles may reflect or even participate in central pathological processes via the gut microbiota-brain axis. While causality cannot be inferred from this cross-sectional data, the consistent association between reduced miR-106a-5p and both systemic (CRP, LDH) and central (MRI) indicators of pathology supports its potential relevance in disease monitoring, although future research should explore whether miR-106a-5p contributes directly to disease mechanisms or merely reflects underlying pathology. Longitudinal studies integrating miRNA profiling, inflammatory biomarker analysis, microbiota composition, and advanced neuroimaging are essential to fully elucidate the role of miR-106a in the pathophysiology of brain–gut communication and neuroinflammatory disorders.

## 4. Discussion

The present study provides evidence of a significant association between reduced gastric juice miR-106a-5p expression and markers of neuroinflammation, systemic injury, vascular dysfunction, and neurodegenerative pathology. Patients presenting neuroimaging abnormalities, including cortical atrophy, lacunar infarctions, and white matter hypodensities, demonstrated lower gastric miR-106a-5p expression compared with healthy controls. In addition, elevated inflammatory biomarkers, particularly CRP and LDH, were associated with significantly higher ΔCt values, corresponding to reduced miR-106a expression. Together, these findings suggest that gastric miR-106a-5p may reflect both systemic inflammatory activity and neurovascular pathology, supporting its potential role as a non-invasive biomarker in neuroinflammatory conditions. Neuroinflammation is increasingly recognized as a central mechanism contributing to the progression of multiple neurodegenerative disorders, including Alzheimer’s disease, vascular dementia, Parkinson’s disease, and ischemic cerebrovascular injury. Persistent activation of microglia and astrocytes, increased oxidative stress, endothelial dysfunction, and disruption of blood–brain barrier integrity contribute to neuronal injury and cognitive decline. Recent evidence suggests that inflammatory and vascular pathways are closely interconnected in neurodegenerative disease progression, particularly in cerebral small vessel disease and chronic ischemic injury [1,2].

MicroRNAs have emerged as important post-transcriptional regulators involved in inflammatory signaling, apoptosis, endothelial homeostasis, oxidative stress responses, and neuronal survival. Among these, miR-106a-5p has attracted increasing attention because of its involvement in pathways associated with vascular injury and neuroinflammation. Du et al. demonstrated that circulating miR-106a-5p levels were significantly altered in patients with acute cerebral infarction and correlated with inflammatory cytokines and neurological severity scores, suggesting that miR-106a-5p may participate in ischemia-associated neuroinflammatory responses [3]. Other studies have linked miR-106a dysregulation to oxidative stress, apoptosis, and neurovascular remodeling, reinforcing its potential relevance in neurodegenerative and cerebrovascular disease [5,6,19].

Our findings extend previous observations by demonstrating that miR-106a-5p is detectable in gastric juice and that its expression correlates with inflammatory biomarkers and structural brain abnormalities. Importantly, the present study introduces gastric juice as a novel and underexplored biofluid for the assessment of neuroinflammatory molecular markers. While most studies investigating miRNAs in neurological disease have focused on plasma or cerebrospinal fluid, gastric juice may provide complementary information regarding gastrointestinal inflammatory activity and gut-derived signaling within the gut–brain axis. The gut–brain axis is increasingly recognized as a complex bidirectional communication network integrating neural, immune, endocrine, metabolic, and microbial signaling pathways. Recent evidence suggests that gut microbiota dysbiosis may contribute to neurodegeneration through chronic immune activation, oxidative stress, endothelial dysfunction, altered blood–brain barrier permeability, and modulation of neuroinflammatory signaling cascades [10,22]. Emerging studies further indicate that microbiota-derived metabolites and inflammatory mediators may influence microglial activation and neuronal homeostasis, thereby contributing to progressive neurodegenerative injury [10,15,22]. Within this framework, gastric-derived miRNAs may represent accessible surrogate markers of systemic inflammatory activity and gut-associated molecular signaling relevant to CNS dysfunction.

Recent studies have strengthened the biological plausibility linking gastrointestinal inflammatory signaling and neurodegeneration. Loh et al. highlighted the important role of the microbiota–gut–brain axis in modulating neuroinflammation and neurodegenerative disease progression through immune, endocrine, and microbial pathways [10]. Similarly, Balakrishnan et al. demonstrated that gut microbiota–immune system interactions may influence neurodegenerative disorders through cytokine-mediated signaling, oxidative stress pathways, and endothelial dysfunction [22]. These findings support the concept that gastrointestinal-derived molecular signals may contribute to systemic inflammatory and neurovascular processes associated with CNS injury.

One important mechanism underlying gut–brain communication may involve extracellular vesicle-mediated transfer of microRNAs and inflammatory mediators. Valadi et al. first demonstrated that exosomes are capable of mediating intercellular transfer of miRNAs between cells [19]. Subsequent investigations showed that extracellular vesicles may transport inflammatory and regulatory miRNAs capable of influencing distant tissues, including the central nervous system [20,21]. Gastric epithelial and immune cells may release miRNA-containing extracellular vesicles in response to local inflammation, oxidative stress, or microbial dysbiosis, thereby contributing to systemic inflammatory signaling. These mechanisms provide biological plausibility for the observed association between gastric-derived miR-106a-5p expression and neurovascular pathology.

Furthermore, miR-106a-5p appears to participate in several pathways relevant to both gastrointestinal and neurological disease, including NF-κB signaling, apoptosis regulation, endothelial homeostasis, and inflammatory cytokine production. Dysregulation of these pathways has been implicated in Alzheimer’s disease, cerebral ischemia, vascular cognitive impairment, and chronic neuroinflammation [1,2,15,21]. Previous research has suggested that miR-106a may influence microglial activation and neuronal apoptosis through PTEN-, STAT3-, and TLR4-associated signaling cascades [3,4,17]. Consequently, reduced miR-106a expression may reflect broader dysregulation of inflammatory and vascular homeostasis rather than a compartment-specific phenomenon.

The observed association between reduced miR-106a-5p expression and MRI abnormalities is particularly noteworthy. Patients with lacunar infarctions and white matter hypodensities demonstrated trends toward lower miR-106a expression, supporting the hypothesis that miR-106a may be linked to cerebral small vessel disease and chronic vascular remodeling. White matter lesions and lacunar infarctions are recognized imaging markers of chronic neurovascular injury and have been strongly associated with cognitive impairment and inflammatory endothelial dysfunction. Although subgroup analyses did not reach statistical significance, likely because of limited sample size and cohort heterogeneity, the overall pattern suggests a potential relationship between miR-106a dysregulation and structural neurovascular damage. Similarly, the inverse association between miR-106a expression and elevated CRP and LDH levels further supports the involvement of miR-106a in inflammatory and tissue injury pathways. CRP represents a well-established marker of systemic inflammation, whereas LDH reflects cellular injury and metabolic stress. Therefore, reduced miR-106a expression may indicate an amplified inflammatory state accompanied by tissue damage and vascular dysfunction. The observed association between elevated cIMT and reduced miR-106a expression additionally supports a possible role for miR-106a in vascular remodeling and endothelial injury. The ROC analysis demonstrated moderate diagnostic performance of gastric miR-106a-5p for distinguishing patients with neurological abnormalities from controls (AUC = 0.701). Although specificity remained modest, the relatively high sensitivity suggests that gastric miR-106a-5p may have value as an exploratory screening or risk stratification biomarker when integrated with inflammatory markers and neuroimaging findings. Importantly, the intention of this study was not to establish superiority over conventional blood-based biomarkers, but rather to investigate whether gastric-derived miRNAs may provide complementary information regarding gut-associated inflammatory signaling relevant to neurodegeneration.

Several limitations should be acknowledged: the cross-sectional design precludes conclusions regarding causality, and the relatively small sample size limits statistical power and generalizability. The heterogeneity of the neurodegenerative group, which included patients with different neurological conditions and MRI abnormalities, may also have introduced variability limiting disease-specific analyses. Furthermore, gastric miRNA expression may be influenced by local gastric conditions, including mucosal inflammation or Helicobacter pylori infection, which were not systematically evaluated. Another important limitation is the use of U6 snRNA as the endogenous normalization control, as its stability in extracellular biofluids remains debated. Future studies should validate gastric juice-specific normalization strategies using stable endogenous miRNAs and spike-in controls. Despite these limitations, the present study introduces gastric juice miR-106a-5p as a promising exploratory biomarker reflecting systemic inflammation, vascular dysfunction, and neurodegenerative alterations. Future research should include larger longitudinal cohorts, standardized normalization approaches, gut microbiota profiling, and multimodal biomarker panels integrating inflammatory markers, circulating miRNAs, and advanced neuroimaging techniques. Such approaches may clarify the mechanistic role of miR-106a-5p within the gut–brain axis and determine its potential clinical utility in neuroinflammatory and neurodegenerative disorders.

## 5. Conclusions

This study demonstrates a significant association between reduced gastric juice miR-106a-5p expression and systemic inflammatory markers, vascular pathology, and MRI-detected neurodegenerative abnormalities. Patients with elevated CRP and LDH levels, increased cIMT, and structural brain changes including cortical atrophy, lacunar infarctions, and white matter hypodensities exhibited lower miR-106a-5p expression compared with controls. These findings support the emerging concept of the gut–brain axis as an important contributor to neuroinflammatory and neurodegenerative processes and suggest that gastric juice may represent a novel and minimally invasive source of molecular biomarkers. Although the diagnostic performance of miR-106a-5p was moderate, its association with inflammatory, vascular, and neuroimaging markers indicates potential value as an exploratory complementary biomarker for identifying individuals at risk of neurovascular and neurodegenerative pathology.

Further longitudinal and mechanistic studies involving larger and more homogeneous patient cohorts are required to validate these findings, clarify causative mechanisms, and determine whether gastric miR-106a-5p may contribute to disease progression or primarily reflect underlying systemic inflammatory dysregulation.

## Figures and Tables

**Table 1:** General characteristics of the patients.

Variable	Control Group (*n* = 16)	Study Group (*n* = 22)	*p*-Value
Age (years)	55 (52–59)	67.5 (61–73)	0.001
miR-106a-5p ΔCt	6.94 ± 3.75	10.68 ± 4.21	0.041
MMSE	24.5 (20–25.5)	19 (10–20)	0.003
Gender			0.132
Male	7 (43.8%)	15 (68.2%)	
Female	9 (56.2%)	7 (31.8%)	
Residency			0.556
Urban	11 (68.8%)	17 (77.3%)	
Rural	5 (31.2%)	5 (22.7%)	
cIMT			0.005
0.9–1.3 mm	11 (68.8%)	5 (22.7%)	
>1.3 mm	5 (31.2%)	17 (77.3%)	
MRI			0.003
Negative	6 (37.5%)	0 (0.0%)	
Positive	10 (62.5%)	22 (100.0%)	
Total Lipids (mg/dL)	550 (400–825)	900 (840–1000)	<0.001
Triglycerides (mg/dL)	160 (140–260)	350 (200–500)	<0.001
HDL (mg/dL)	43 (42–48.5)	44 (40–50)	0.737
LDL (mg/dL)	149 (134.5–177)	178.5 (167–190)	0.002
C-reactive protein (mg/L)	2.5 (2–3)	23.5 (17–32)	<0.001
ALP (U/L)	118 ± 22	165 ± 36	0.018
LDH (U/L)	152.5 (139–168)	270 (240–300)	<0.001

## Data Availability

The data presented in this study are available on request from the corresponding author. (The data are not publicly available due to ethical restrictions).
